# National Basketball Association combine scores as a predictive measure of lower limb surgery over 10 consecutive seasons (2010–2020): A retrospective review

**DOI:** 10.1002/jeo2.70336

**Published:** 2025-07-07

**Authors:** Ravi A. Patel, Rohan M. Shah, Tyler M. Hauer, Michael A. Terry, Vehniah K. Tjong

**Affiliations:** ^1^ Northwestern University Feinberg School of Medicine Chicago Illinois USA; ^2^ Department of Orthopaedic Surgery University of Toronto Toronto Ontario Canada; ^3^ Department of Orthopaedic Surgery Northwestern Medicine Chicago Illinois USA

**Keywords:** knee arthroscopy, lower limb surgery, NBA Combine, preventative sports medicine

## Abstract

**Purpose:**

The purpose of this study was to correlate National Basketball Association (NBA) Combine scores with future surgical lower limb injury to determine if NBA Combine scores can be predictive of future surgery on the lower limb.

**Methods:**

A retrospective review of NBA surgical lower limb injuries was performed using a data set covering 10 consecutive NBA seasons (2010–2020). All NBA Combine data were obtained through the official NBA Combine website. NBA Combine data were matched to injury list and compared against noninjured control, described using means and standard deviations. Differences were evaluated using independent *t*‐tests, with an a priori level of significance at *p* < 0.05.

**Results:**

A total of 27,105 injury transactions were reported and a total of 130 players were identified who had undergone lower limb surgical management. There was no statistically significant difference in anthropometric stats (weight, body fat % and height). Lane agility time, three quarter sprint and max bench press showed no statistically significant differences. However, standing vertical leap and max vertical leap showed statistically significant differences, with values increased in injured group. Mean standing vertical was 73.86 cm (SD = 7.82) in noninjured and 76.00 cm (SD = 7.77) in the injured group. Mean max vertical was 86.89 cm (SD = 9.37) in noninjured and 89.31 cm (SD = 9.17) in injured group. Knee injuries (80.0%) were most prevalent, followed by ankle (8.5%), calf (7.7%), and thigh (3.8%). Knee surgeries comprised of general surgery on knee (42.3%), meniscal surgery (20.2%), arthroscopic knee surgery (18.3%), anterior cruciate ligament reconstruction (15.4%), and patellar tendon repair (3.8%).

**Conclusions:**

Increased NBA Combine scores of standing and maximum vertical leap may be related to future lower limb injury requiring surgical management among basketball players. The knee remains the most injured joint with the majority of knee surgeries performed arthroscopically addressing meniscal pathology.

**Level of Evidence:**

Level III.

**Abbreviations**
ACL: anterior cruciate ligament
AE: athletic exposure event
NBA: National Basketball Association
NFL: National Football League
OR: odds ratio
PT: physical therapy

## INTRODUCTION

The National Basketball Association (NBA) is a sports business predicated on winning basketball games. Team performance, key players and championships play a critical role in increasing value for NBA franchises [19]. Unfortunately, injuries to NBA players lead to missed games and have been shown to lower team performance [15]. When acquiring players to build a team, general managers must factor in injury risk to both increase team performance and franchise value.

The NBA Draft is arguably the most important way teams acquire players. Drafting a player allows teams to own the rights to sign that player at the beginning of their career. Even more crucial, if general managers draft superstar players, these teams can then sign their players to long‐term contracts due to rookie super‐max contract extensions. These deals allow teams to retain home‐grown superstars while retaining financial and roster flexibility, increasing their chances of being successful on the court and commercially [8].

However, drafting injury‐prone players can negatively affect the development of aspiring teams, seen most visibly through the 2010s Chicago Bulls and Derrick Rose. Especially in the setting of surgical injuries, this may have a significant impact on a player's ability to compete at the professional level or even return to sport.

The NBA Draft Combine is an athletic testing event prior to the NBA Draft, showcasing prospect athleticism through combine scores. Combine tests measure anthropometric variables (height, standing reach, weight, etc.) and strength/agility. Strength and agility tests include lane agility time, shuttle run, three quarter sprint, standing vertical leap and max vertical leap. Although combine tests are supposed to help general managers draft higher performing players, research has shown that the current measurements fail to offer meaningful performance data on the value these players add to their respective teams [4]. As the NBA Combine does test athleticism, we questioned why combine metrics have not been used to predict injury susceptibility. If combine scores do predict injury risk, it could be a valuable metric for NBA teams when selecting future talent.

To our knowledge, there has been no previous data correlating NBA Combine scores with lower limb injury susceptibility. The purpose of this study was to correlate NBA Combine scores with future surgical lower limb injury to determine if NBA Combine scores can be predictive of future surgery on the lower limb (including knee and ankle). This information may be used by the NBA to increase team value and performance through more reliable drafting. In addition, it can be used by team physicians and athletic training staff to focus on specific injury prevention in these players once risk is identified.

## METHODS

A retrospective review of NBA surgical lower limb injuries was performed using a data set covering 10 consecutive NBA seasons (2010–2020) [9, 14]. The data set, uploaded to the website Kaggle, is scraped from the website Pro Sports Transactions. Pro Sports Transactions is a public database documenting all sports transactions across the five major North American Sports. The data set used in this study identified and documented each injury transaction in the NBA from 2010 to 2020. Out of this study, we screened for lower limb surgical procedures. The process of compiling an injury list from publicly available team transaction reports is similar to the previous research methodology of professional sports injuries [1, 2, 11, 18, 20]. Inclusion criteria included transactions involving injury below the hip requiring surgery. Exclusion criteria included injury above or at the hip, nonsurgical treatment of injury and repeat transactions regarding the same discrete injury. The data were screened using keywords to pull out injury transactions pertaining to lower limb surgery. This included but was not limited to procedures involving torn meniscus, torn anterior cruciate ligament (ACL), torn Achilles, torn patella tendon, torn hamstring, arthroscopic knee surgery and arthroscopic ankle surgery.

All NBA Combine data were obtained through the official NBA Combine scores website, which is publicly available [7]. To match the combined data with injured players, we individually searched for each player included in our injury list. All draft combine strength and agility tests were recorded, including lane agility time, shuttle run, three quarter sprint, standing vertical leap and max vertical leap. Specific draft combine anthropomorphic data were also included, consisting of height without shoes, standing reach, weight and wingspan. The NBA Combine data stretches from 2000 to 2023. Therefore, only players that were drafted after 2000 were included in the study. Furthermore, players that declined to participate in the combine tests were excluded due to the inability to match their combine data with their injury. A control group was created as a standard to compare the injured players' combine data against. The control group included all NBA Combine participants except players who were identified to have undergone lower limb surgery. The control group's combined scores were then matched by individual and reported, congruent with our data collection of the injured population.

### Data Analysis

Combine data were described using means and standard deviations, calculated for both the injured and control populations. Differences between the injured and uninjured were evaluated using independent *t*‐tests, with an a priori level of significance at *p* < 0.05. Data were recorded using a similar method to previously published research using NBA Combine data and injuries [12].

## RESULTS

**Figure 1:**
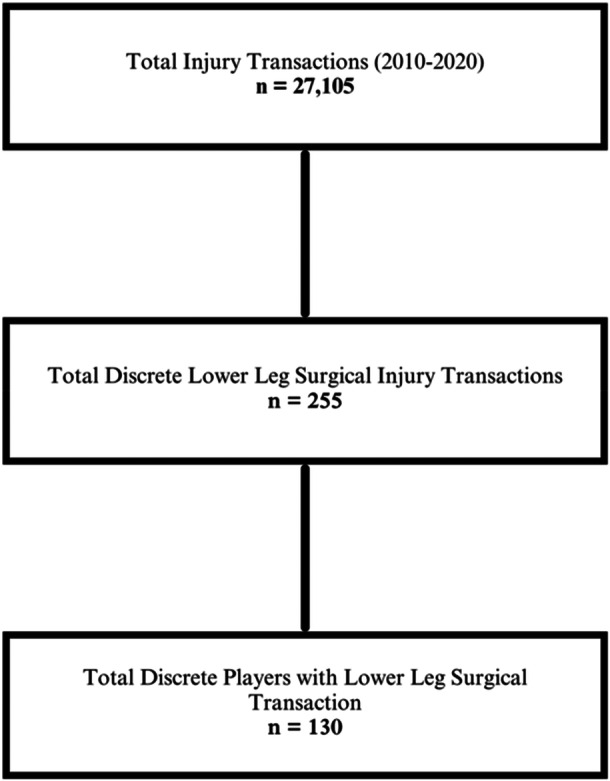
Injured player inclusion flow chart.

A total of 27,105 injury transactions were reported across the data set. The search identified 255 discrete injury transactions involving lower limb surgical management. Excluding players with multiple discrete injuries, a total of 130 players between 2010 and 2020 were identified who had undergone lower limb surgical treatment, composing 9.6% of NBA Combine participants between 2000 and 2020 (Figure 1). The control group consisted of 1220 players who participated at the NBA Combine and did not undergo lower limb operation. There was no statistically significant difference in anthropometric stats (weight, body fat % and height), suggesting positional and bodily variability did not affect the distribution between the noninjured and injured groups. Strength and agility stats, lane agility time, three quarter sprint and max bench press showed no statistically significant difference between groups. However, standing vertical leap and max vertical leap showed statistically significant differences between groups, with values increased in players that were injured and surgically managed. The mean standing vertical was 73.86 cm (SD = 7.82) in the noninjured group and 76.00 cm (SD = 7.77) in the injured group. The mean max vertical was 86.89 cm (SD = 9.37) in the noninjured group and 89.31 cm (SD = 9.17) in the injured group (Table 1).

**Table 1:** Complete multivariate regression results.

Characteristic	Noninjured group^a^	SD^b^	Injured group	SD	*p* value
Anthropometric stats					
Weight (kg)	97.96	12.07	99.23	11.86	0.254
Body fat %	7.66	2.93	7.51	3.12	0.602
Height (cm)	192.13	32.00	196.55	18.82	0.123
Strength and agility stats					
Lane agility time (s)	11.45	0.62	11.34	0.52	0.08
Three quarter sprint (s)	3.29	0.13	3.28	0.13	0.33
Standing vertical (cm)	73.86	7.82	76.00	7.77	0.005*
Max vertical (cm)	86.89	9.37	89.31	9.17	0.009*
Max bench (reps)	10.69	5.28	10.80	5.09	0.85

*Statistically significant (*p* < 0.05).

^a^ Mean of variable.

^b^ SD, standard deviation.

**Figure 2:**
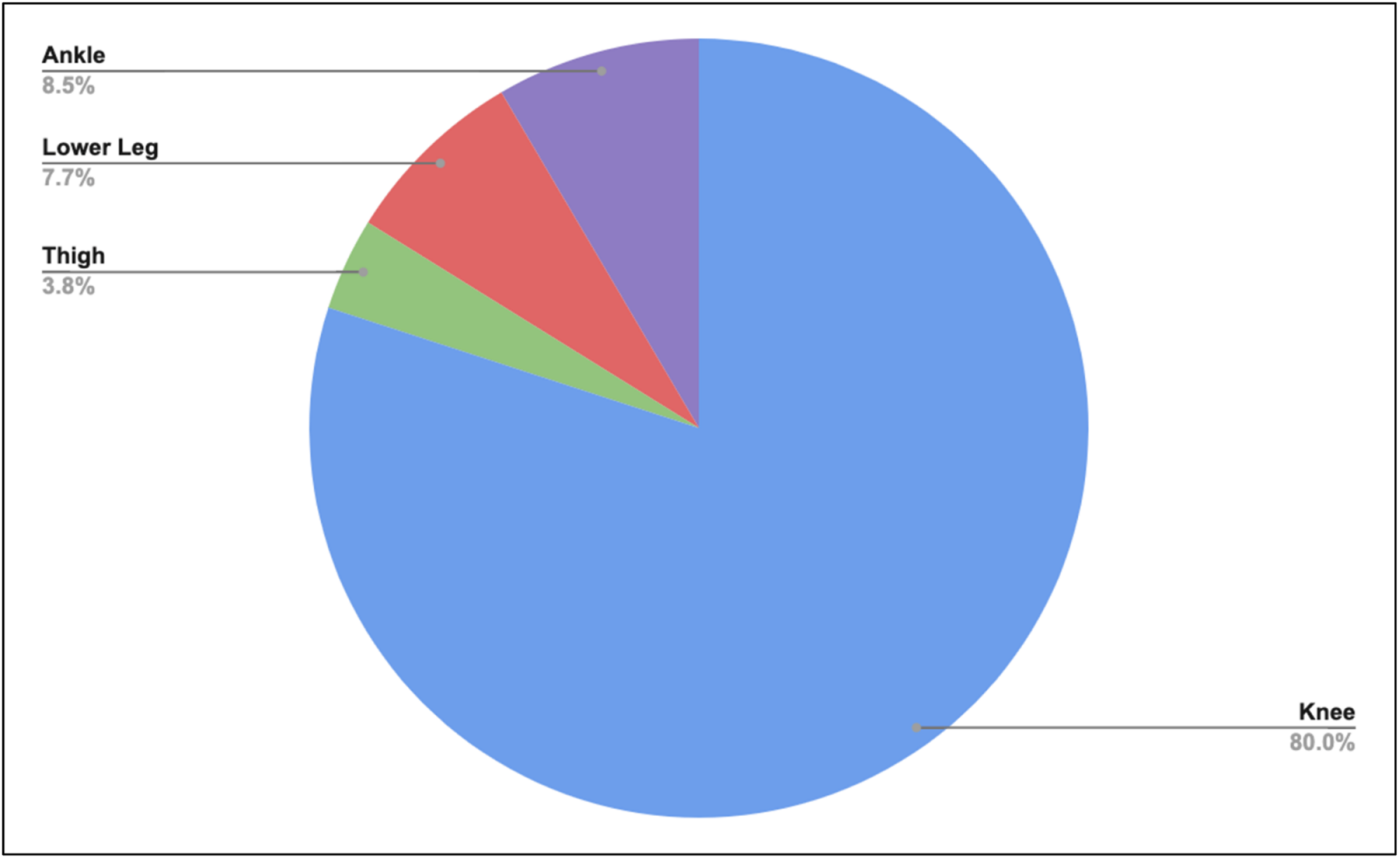
Injury by location.

Separating lower limb injury by location, knee injuries (80.0%) were the most prevalent, followed by ankle (8.5%), calf (7.7%) and thigh (3.8%) (Figure 2).

**Figure 3:**
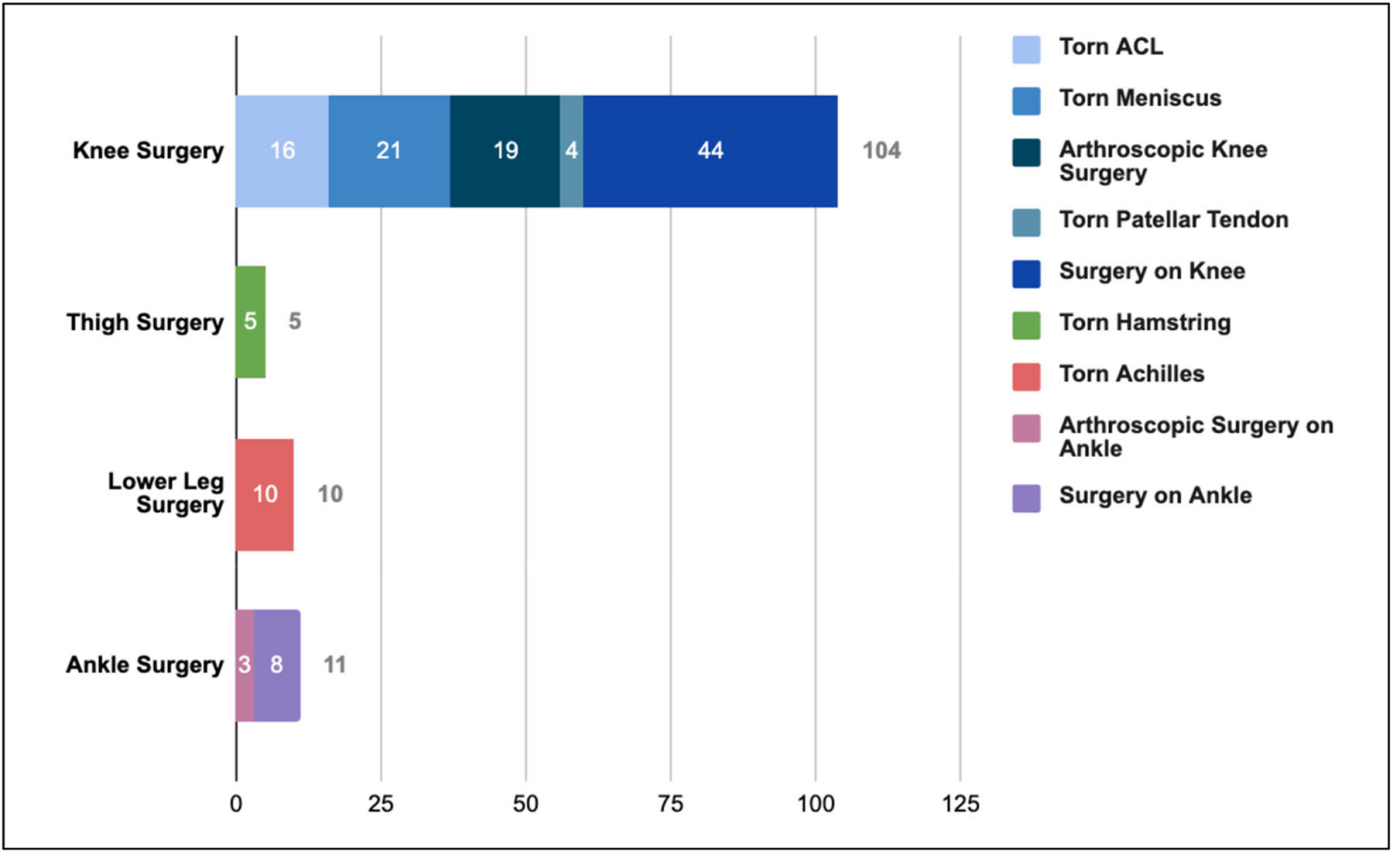
Injury by procedure.

Analysing injuries by procedure/description within injury location, knee injuries comprised of general surgery on knee (42.3%), meniscal surgery (20.2%), arthroscopic knee surgery (18.3%), ACL reconstruction (15.4%) and patellar tendon repair (3.8%). Thigh surgeries were solely composed of hamstring repair (100%). Similarly, all calf surgeries were Achilles tendon repair (100%). Ankle surgery consisted of surgery on ankle (72.7%) and arthroscopic surgery on ankle (27.3%) (Figure 3).

## DISCUSSION

NBA Combine scores are important in that they provide a useful metric for understanding athleticism of NBA athletes and are standardised across the majority of players entering the league. Professional‐level basketball players who underwent lower limb surgery in their career showed statistically significant differences in standing and max vertical leap scores in the NBA Combine compared to noninjured NBA players across the same time span. Our research is the first to correlate NBA Combine scores with surgical injury within elite basketball athletes.

Although NBA Combine scores as a predictive measure have not been previously studied, other investigations into the NBA Combine and health have been conducted. Mehran et al. analysed NBA Combine scores after ACL reconstruction and found no statistically significant difference in combine performance between 21 ACL‐reconstructed athletes and a matched control cohort [13]. Interestingly, there was a trend in standing vertical leap as the ACL reconstruction group had a lower mean standing vertical leap (71.6 cm) than control (74.2 in), although this trend did not reach statistical significance. In another study, Khalil et al. found that there was no statistically significant difference in combine performance between 12 players who had undergone a partial meniscectomy and a matched control cohort [12]. The study did find statistically significant positive correlation between months after surgery and standing vertical leap (Spearman correlation = 0.690) and max vertical leap (Spearman correlation = 0.650) as well as statistically significant negative correlation between months after surgery and agility (Spearman correlation = −0.596). These studies demonstrate NBA Combine scores have been successfully used as a proxy for surgical outcome success and as a research metric. They also confirm injury prior to the NBA Combine does not impact the scores and predictive value of our study.

The literature consistently indicates lower limb surgery offers minimal change in NBA Combine scores, yet no research has investigated the predictive capability of NBA Combine scores in injury prevention. In the NFL, Brophy et al. describe a predictive measure of career longevity through NFL Combine assessment [5]. The research group graded each prospect through an orthopaedic grade and found the grade correlated with probability of playing in the National Football League (NFL). Fifty‐eight percent of players with a high grade and 55% of players with a low grade played at least one game in the NFL, whereas only 36% of players with a failing grade played a game in the NFL. Players with a higher grade played on average more games in the NFL (41.5 games) compared to low grade (34.2 games) and failing grade (19.2 games). Of note, they found players with meniscal injury and ACL injury to be less likely to play in the league than players without these diagnoses. By focusing on NBA Combine scores and their predictive value upon injury, we provide a novel way to understand athleticism and injury in high level basketball players. Our study demonstrates that standing and max vertical leap are statistically different in NBA players that retrospectively underwent lower leg surgery.

Understanding the mechanism behind this difference requires future research, including analysing the biomechanics underpinning a basketball jump. Schiltz et al. found relative isokinetic and functional performances of professional basketball players to be similar to junior players through weight‐adjusted physical therapy (PT) values, suggesting elite vertical jump ability does not influence physical energy requirements of the leg [17]. In contrast, players with a history of knee injury displayed abnormal isokinetic profile. This suggests our findings may point to a different mechanism of injury than increased load upon the lower leg due to jumping ability. In a separate study, Cassinat et al. determined more minutes per game (odds ratio [OR] = 1.13), greater usage rate (OR = 1.02), centre position (OR = 1.64) and lower player efficiency rating (OR = 0.96) to be associated with lower extremity surgical intervention [6]. Pairing NBA Combine scores with specific playstyle of each athlete may provide greater detail into the mechanism of injury in players with a higher vertical leap.

A secondary finding in our research was the distribution of location in lower leg injury requiring surgery. We found knee injury (80% of total) to comprise the majority of surgical intervention in our sample, followed by ankle (8.5%), lower leg (7.7%) and thigh (3.8%). Andreoli et al. found knee and ankle injuries were the most injured body parts across all age and skill levels of basketball [3]. In the NBA, Cassinat et al. found a higher incidence of knee surgical intervention (0.23 per 1000 athletic exposure events [AE]) than ankle injuries (0.04 AE) across 1153 ankle and knee injuries [6]. Although more specific recommendations cannot be made due to a large portion of surgery falling under ‘surgery on knee’, Hull et al.'s video‐based analysis found direct trauma, cutting, and landing as the scenarios in which knee injury occurs most frequently [10]. Direct trauma events are difficult to predict; however, athletic trainers and medical specialists may emphasise increased strength and flexibility throughout the knee in athletes with an elevated vertical leap to help reduce injury from cutting and landing. In female basketball athletes with valgus alignment, Rostami et al. used a training program, STOP‐X, to improve balance and knee valgus alignment; adapting this program or a program similar to it into professional male basketball athletes with elevated vertical leap may help prevent knee injury in the NBA [16]. Outside of the knee, strength and flexibility in the posterior leg is also necessary, as evidenced by the proportion of injuries related to torn hamstrings (3.8%) and torn Achilles (8.5%).

Limitations of this study include data availability, as the discrepancy between NBA Combine participation (2000–2020) and injury dataset (2010–2020) does not include players drafted after 2000 that suffered a surgical lower limb injury in the 2000s. Another challenge with the dataset is the lack of consistency within NBA injury transaction reporting. Injuries are reported to the public through team press releases. The description of each injury is included in these reports; unfortunately, they often do not provide specific medical detail. Still, we included this information as it provides insight into injuries such as torn ACL and torn meniscus. Specific medical procedures could not be delineated in some cases, for instance, reporting ‘surgery on knee’ instead of ‘surgery on torn lateral meniscus.’ The lack of clarity of procedure makes it more difficult to implement specific preventive rehabilitative measures for these players.

## CONCLUSIONS

Certain NBA Combine scores such as standing and max vertical leap may be predictive of future lower limb injury requiring surgery. Further research is required to strengthen the relationship between NBA Combine measurements and injury, including analysing playstyle in conjunction with combine scores. Prospective research on the implementation of player specific training to limit injuries associated with elevated vertical leap may aid in individual career longevity and greater team success.

## AUTHOR CONTRIBUTIONS

All authors contributed to the study conception and design. Material preparation, data collection and analysis were performed by Ravi Ameet Patel, Rohan M. Shah and Tyler M. Hauer. The first draft of the manuscript was written by Ravi Ameet Patel and all authors commented on previous versions of the manuscript. All authors read and approved the final manuscript.

## CONFLICT OF INTEREST STATEMENT

The authors declare no conflicts of interest.

## ETHICS STATEMENT

This is a retrospective study of publicly available data. There is no ethical approval required.

## Data Availability

The data that support the findings of this study are openly available at https://www.nba.com/stats/draft/combine-strength-agility and https://www.kaggle.com/datasets/ghopkins/nba-injuries-2010-2018.
